# *Actinobaculum schaalii *- invasive pathogen or innocent bystander? A retrospective observational study

**DOI:** 10.1186/1471-2334-11-289

**Published:** 2011-10-26

**Authors:** Sarah Tschudin-Sutter, Reno Frei, Maja Weisser, Daniel Goldenberger, Andreas F Widmer

**Affiliations:** 1 Division of Infectious Diseases and Hospital Epidemiology, University Hospital, Basel, Switzerland; 2 Division of Clinical Microbiology, University Hospital, Basel, Switzerland

## Abstract

**Background:**

*Actinobaculum schaalii *is a Gram-positive, facultative anaerobic coccoid rod, classified as a new genus in 1997. It grows slowly and therefore is easily overgrown by other pathogens, which are often found concomitantly. Since 1999, *Actinobaculum schaalii *is routinely investigated at our hospital, whenever its presence is suspected due to the detection of minute grey colonies on blood agar plates and negative reactions for catalase. The objective of this study was to determine the clinical significance of *Actinobaculum schaalii*, identified in our microbiology laboratory over the last 11 years.

**Methods:**

All consecutive isolates with *Actinobaculum schaalii *were obtained from the computerized database of the clinical microbiology laboratory and patients whose cultures from any body site yielded this pathogen were analyzed. Observation of tiny colonies of Gram-positive, catalase-negative coccoid rods triggered molecular identification based on 16S rRNA gene sequencing.

**Results:**

40 isolates were obtained from 27 patients during the last 11 years. The patient's median age was 81 (19-101) years, 25 (92.6%) had underlying diseases and 12 (44.4%) had a genitourinary tract pathology. *Actinobaculum schaalii *was isolated in 12 urine cultures, 21 blood cultures, and 7 deep tissue biopsies. Twenty-five (62.5%) specimens were monobacterial, the remaining 15 (37.5%) were polybacterial 7/7 deep tissue samples (three bloodcultures and five urine cultures). Recovery from urine was interpreted as colonization in 5 (18.6%) cases (41.6% of all urine samples). Six (22.2%) suffered from urinary tract infections, six (22.2%) from abscesses (skin, intraabdominal, genitourinary tract, and surgical site infections) and 10 (37.0%) from bacteremia.

**Conclusions:**

In this largest case series so far, detection of *Actinobaculum schaalii *was associated with an infection - primarily sepsis and abscesses - in 81.5% of our patients. Since this pathogen is frequently part of polymicrobial cultures (42.5%) it is often overlooked or considered a contaminant. Detection of *Actinobaculum schaalii *in clinical isolates mainly reflects infection indicating that this Gram-positive rod is not an innocent bystander.

## Background

*Actinobaculum schaalii *is a Gram-positive, facultative anaerobic, nonmotile coccoid rod, classified as a new genus in 1997 [1]. The genus *Actinobaculum *includes *A. schaalii, A. suis, A. massiliae*, and *A. urinale *and is closely related to the genera *Actinomyces *and *Arcanobacterium *[1].

*Actinobaculum schaalii *grows slowly after 48 h in an anaerobic atmosphere at 37°C as tiny grey colonies, less than 1 mm in diameter, and shows weak β-hemolysis on agar plates containing 5% horse or sheep blood. It is catalase, oxidase, and urease negative and is easily overgrown by other bacteria, which are often found concomitantly. Because of its slow anaerobic growth and resemblance to the normal bacterial flora on skin and mucosa, *A*. *schaalii *is often overlooked or considered a contaminant. These difficulties regarding detection and identification impede evaluation of the clinical impact of this pathogen and of its potential to cause invasive infection.

*A. schaalii *has been reported to be responsible for urinary tract infections, mainly in elderly patients with underlying urological predispositions [2-5]. It has also been recovered from other human clinical specimens than urine such as blood, but its pathogenic potential remains unknown.

Since 1999, *A. schaalii *is routinely screened at our hospital. The objective of this study was to determine the clinical significance of *A. schaalii*, identified in our microbiology laboratory over the last 11 years.

## Methods

### Setting

The University Hospital of Basel is an 855 bed tertiary care center in Basel, Switzerland, with approximately 30'000 admissions per year. The study was approved by the local ethics committee as part of the quality assurance program.

### Patients and data collection

All consecutive isolates with *A. schaalii *were obtained from the computerized database of the clinical microbiology laboratory and patients whose cultures from any body site yielded this pathogen were analyzed. A board certified infectious diseases specialist then reviewed the medical records of these patients and collected data regarding patient demographic characteristics, underlying diseases or condition, clinical manifestations at the time of detection of *A. schaalii*, antibiotic therapy received, and clinical outcome. Definite antibiotic treatment was defined as the antibiotic regimen chosen after identification of the pathogen was completed. Presence of *A. schaalii *was categorized into colonization and infection. Urinary tract infections, surgical site infections, bloodstream infections, bone and joint infections, intraabdominal infections, skin and soft tissue infections, and upper and lower respiratory tract infections were defined according to the criteria of the Centers for Disease Control and Prevention (CDC) [6].

### Culture and Species Identification of Actinobaculum schaalii

Grey, tiny colonies with weak β-hemolysis or without hemolysis on 5% Columbia sheep blood agar after 48 h of anaerobic or 5% CO_2 _incubation were further analyzed. All isolates showing Gram-positive coccoid rods and a negative catalase reaction were identified by partial 16S RNA gene sequencing using Microseq 500 Bacterial Identification Kit (Applied Biosystems, Rotkreuz, Switzerland) [7-9]. All bacteria growing in specimens supposed to be sterile and monocultures from normally unsterile bodysites were considered to be clinically relevant.

Blood cultures performed at the University Hospital always involve cultivation in an aerobic and in an anaerobic bottle.

## Results

40 specimens with detection of *A. schaalii *were obtained from 27 different patients during the last 11 years. The patient's median age was 81 (19-101) years, and there was a slight male predominance (59.3%). Twenty-five patients (92.6%) had underlying diseases, of which cardiopathy was most commonly encountered (14, 51.9%). Twelve patients (44.4%) had a pathology of the genitourinary tract, mostly prostatic hyperplasia (5, 18.5%) and five patients (18.5%) had an urinary catheter or a double- J-catheter in place at the time of detection of *A*. *schaalii*. Only one patient (3.7%) was receiving immunosuppressive treatment due to renal transplantation (Table 1).

**Table 1:** Baseline characteristics and outcome

Patient's baseline characteristics (n = 27) and outcome	
**Age (years)**	
Mean	73
Median	81
Range	19-101
**Gender**	
Male	16 (59.3%)
Female	11 (40.7%)
**Hospitalstay (days)**	
Mean	18
Median	9
Range	1-79
**Immunosuppression**	**1 (3.7%)**
**Comorbidities**	**25 (92.6%)**
Cancer	7 (25.9%)
Cardiopathy	14 (51.9%)
Chronic renal insufficiency	4 (14.8%)
Diabetes mellitus	4 (14.8%)
Neurological disorder	13 (48.1%)
Hypertension	6 (22.2%)
Chronic pulmonary disorder	2 (7.4%)
Chronic Vascular disorder	4 (14.8%)
Endocriological disorder (other than diabtes mellitus	3 (11.1%)
Chronic pancreatitis	1 (3.7%)
**Underlying genitourinary tract pathology**	**12 (44.4%)**
Nephrolithiasis	1 (3.7%)
Stenosis	4 (14.8%)
Double-J catheter	2 (7.4%)
Urinary catheter	3 (11.1%)
Prostatic hyperplasia	5 (18.5%)
Renal cysts	1 (3.7%)
Uterine and vaginal prolapse	1 (3.7%)
Renal transplantation	1 (3.7%)
**Outcome**	
Discharge	17 (63.0%)
Referal to another hospital	3 (11.1%)
Referal to a longterm-care facility	5 (18.5%)
Death	2 (7.4%)

*A. schaalii *was detected in 21 blood cultures, 7 deep tissue biopsies and 12 urine cultures. Its presence was interpreted as colonization of the urinary tract without clinical signs or symptoms of infection in five (18.5%) patients, accounting for 41.6% of all urine samples. Six patients (22.2%) suffered from urinary tract infections, six (22.2%) from abscesses (skin, intraabdominal, genitourinary tract, and surgical site infections) and 10 (37.0%) from bacteremia (Table 2).

**Table 2:** Distribution of infections and colonisation with *Actinobaculum schaalii*

Infections and colonisation with *Actinobaculum schaalii *(n = 27)	
**Bacteremia**	**10 (37.0%)**
with urinary tract infection	5 (18.5%)
with intaabdominal infection	3 (11.1%)
with spondylodiscitis	1 (3.7%)
with pneumonia	1 (3.7%)
	
**Abscess**	**6 (22.2%)**
Abdominal	1 (3.7%)
Skin	1 (3.7%)
Surgical site infection (upper jaw and after bladder-stone extraction)	2 (7.4%)
Genitourinary tract	2 (7.4%)
	
**Urinary tract infection**	**6 (22.2%)**
with detection of *A. schaalii *in urine culture	6 (22.2%)
	
**Colonisation (detection of *A. schaalii *in urine without clinical signs of infection)**	**5 (18.6%)**

*A. schaalii *affected the genitourinary tract infections in most cases (48.1%). The majority of patients (10/13, 76.9%) affected had underlying pathologies of the urinary tract. In three patients with positive blood cultures, diagnosed as urosepsis, Gram-positive rods (10^4^-10^6 ^colonies per ml) were detected in urine cultures, however, weren't further identified as these cultures were polymicrobial. Abscesses of the genitourinary tract involved the scrotum in one patient and the renal pelvis in another (Table 2).

Surgical site infections occurred in two patients, one after an operation involving the upper jaw, the other after bladder-stone extraction.

Bacteremia with *Actinobaculum schaalii *was associated with spondylodiscitis and pneumonia, in one case respectively, with intraabdominal infection in three cases and with urinary tract infections in 5 cases (Table 2). In the patient with spondylodiscitis, *Actinobaculum schaalii *was also detected in the CT-guided biopsy of the disc. Underlying intraabdominal infections detected were occlusive ileus and diverticulitis.

Twenty-three (57.5%) specimens were monobacterial, the remaining 17 (42.5%) were polybacterial 7/7 deep tissue samples, five blood cultures, and five urine cultures (Table 3). In patients with polybacterial blood cultures, the other pathogens detected were *Pseudomonas aeruginosa *(*P. aeruginosa *in 2 of 2 and *A. schaalii *in 1 of 2 blood cultures) in a patient with urosepsis, *Finegoldia magna *(*Finegoldia magna *in 2 of 2 and *A. schaalii *in 4 of 4 bloodcultures) in a patient with diverticulitis, *Clostridium clostridiforme *and *Bacteroides fragilis *(both in 1 of 4 and *A. schaalii *in 1 of 4 blood cultures) in a patient with obstructive ileus and *Veillonella *(in 2 of 8 and *A. schaalii *in 8 of 8 bloodcultures) in a patient with unclear intraabdominal infection. Of interest, the patient with *P. aeruginosa *and *A. schaalii *in the blood culture, also had Gram-positive rods (10^4 ^CFU/ml) and Gram-negative rods (10^5 ^CFU/ml) in the urine culture, however, these weren't further differentiated.

**Table 3:** Distribution of monobacterial and polybacterial isolates of *Actinobaculum schaalii*

Specimens with detection of *Actinobaculum schaalii *(n = 40)	
monobacterial	23 (57.5%)
polybacterial	17 (42.5%)
	
**Urine**	**12**
monobacterial	7 (58.3%)
polybacterial	5 (41.7%)
	
**Blood**	**21**
monobacterial	16 (76.2%)
polybacterial	5 (23.8%)
	
**Abscess**	**7**
monobacterial	0 (0.0%)
polybacterial	7 (100.0%)

Six patients received no antibiotic treatment, in five of which detection of *A. schaalii *was interpreted as colonization. The remaining patient suffered from an abscess involving the skin, which was surgically drained. Most patients were treated with amoxicillin/clavulanic acid (10, 37.0%) followed by administration of a quinolone (7, 25.9%).

Median duration of antibiotic treatment was 15.5 days (range 3 to 84 days). The majority of patients could be discharged (17, 63.0%), two (7.4%) patients died due to metastatic carcinoma, the remaining were transferred to another hospital (3, 11.1%) or to a long-term-care facility (5, 18.5%).

## Discussion

*A. schaalii *caused an invasive infection in 81.5% of our patients and its presence was interpreted as colonization of the urinary tract in five patients. Infections of the genitourinary tract were by far the most common manifestation of invasive disease. The ability of this pathogen to cause urinary tract infections has also been reported in the literature previously. Beguelin et al. described the clinical features of 20 patients and found urinary tract infections to be the most common manifestation (11/20, 55%) [10]. The authors further described a high rate of underlying urinary tract pathology as detected in our study. We detected *A. schaalii *in the blood cultures of 10 patients, only half of which were associated with urinary tract infections. The majority of the remaining patients had underlying intraabdominal infections and one patient suffered from spondylodiscitis. Therefore, detection of this pathogen in blood cultures should prompt the clinician to search also for abdominal or other foci if no association with the genitourinary tract can be found.

Beguelin et al. described the results of antimicrobial resistance testing in their study, although there are no recognized minimal inhibitory concentration (MIC) interpretative standards for *A. schaalii *[10]. They found that most of their strains showed high MIC levels to ciprofloxacin (>32 mg/L) and to trimethoprim/sulfamethoxazole (>32 mg/L), however, all of their 20 isolated tested, showed low MIC level to beta-lactams (≤ 0.19 for penicillin) [10]. Similar results were described by Cattoir et al. after testing 48 clinical isolates [11]. The majority of our patients were treated with betalactam-antibiotics, seven however, all with urinary tract infections, were treated with quinolones, possibly without in vitro activity. We therefore conclude, that susceptebilty testing should be routinely performed, when *A. schaalii *is detected in relevant clinical specimens and interpretative standards for interpretation of MICs should be developed. Empirically initiated treatment for urinary tract infections often consists of quinolones or trimethoprim/sulfamethoxazole and may therefore, not be effective.

*A. schaalii *was often detected together with other bacteria (17 of 40 cultures, 42.5%), especially in urine cultures (5 of 12, 41.7%) and in all abscesses (7 of 7, 100%). In contrast, Beguelin et al. described a much lower rate of polybacterial cultures, with 18 out of 21 (86%) specimens being monobacterial [10]. As this pathogen is difficult to detect in the laboratory and is easily overgrown by other bacteria, awareness of its existence is crucial as not to underestimate its presence. Bank et al. analyzed 252 urine samples for presence of *A. schaalii *by a real- time quantitative PCR they developed and found 41 of 252 samples (16%) to be positive with bacterial concentrations >10^4 ^CFU/mL [3]. Of 155 urine samples from patients >60 years of age, 34 (22%) were PCR positive, of which 31 (91%) - the vast majority - harbored other common uropathogenic bacteria, in addition to *A*. *schaalii*. In our study three patients with *A. schaalii *in their blood cultures had Gram-positive rods (10^4^-10^6 ^colonies per ml) in urine cultures drawn at the same time, however, these weren't further identified as cultures were polymicrobial. One of the patients described in our study with *P. aeruginosa *and *A. schaalii *in blood cultures, also had Gram-positive rods (10^4^/ml) and Gram-negative rods (10^5^/ml) in the urine culture drawn at the same time. It is plausible that the pathogens detected in the urine culture, were *A. schaalii *and *P. aeruginosa*, respectively, demonstrating that *A. schaalii *has the ability to invade the bloodstream even in the presence of a well-known uro-pathogen as *P. aeruginosa*.

As our data demonstrate that this pathogen invaded the bloodstream in the presence of other bacteria in primary foci, we conclude that *A. schaalii *is not just an innocent bystander, but has the potential to become an invasive pathogen, leading to severe invasive infection as sepsis, spondylodiscitis [12], and endocarditis [13].

## Conclusions

In this largest case series so far, detection of *Actinobaculum schaalii *was associated with an infection - primarily sepsis and abscesses - in 81.5% of our patients. Since this pathogen is frequently part of polymicrobial cultures (42.5%) it is often overlooked or considered a contaminant. Detection of *Actinobaculum schaalii *in clinical isolates mainly reflects infection indicating that this Gram-positive rod is not an innocent bystander.

## Competing interests

The authors declare that they have no competing interests.

## Authors' contributions

All authors read and approved the final manuscript. ST-S wrote the manuscript and analyzed the data RF performed and supervised the microbiological analyses. MW supervised and edited the manuscript. DG performed and supervised the microbiological analyses. AFW initiated the study, analysed the data, supervised the whole project and edited the manuscript.

## Pre-publication history

The pre-publication history for this paper can be accessed here:

http://www.biomedcentral.com/1471-2334/11/289/prepub
